# Dietary Magnesium Intake Level Modifies the Association Between Vitamin D and Insulin Resistance: A Large Cross-Sectional Analysis of American Adults

**DOI:** 10.3389/fnut.2022.878665

**Published:** 2022-06-07

**Authors:** Ya Liu, Rongpeng Gong, Haixiu Ma, Siai Chen, Jingwei Sun, Jiarui Qi, Yidan Pang, Juan An, Zhanhai Su

**Affiliations:** ^1^Department of Basic Medical Sciences, Qinghai University Medical College, Xining, China; ^2^Key Laboratory for High Altitude Medicine, Research Center for High Altitude Medicine, Xining, China

**Keywords:** vitamin D, cross-sectional studies, dietary magnesium intake, insulin resistance, American adults

## Abstract

**Background:**

Previous clinical studies and randomized controlled trials have revealed that low serum vitamin D levels are associated with the risk of developing insulin resistance. Magnesium has been reported to be a protective factor for insulin resistance, and magnesium has been considered an important co-factor for vitamin D activation. However, the effect of dietary magnesium intake on the relationship between vitamin D and the risk of developing insulin resistance has not been comprehensively investigated. Therefore, we designed this cross-sectional analysis to assess whether dietary magnesium intake modifies the association of vitamin D and insulin resistance.

**Methods:**

A total of 4,878 participants (male: 48.2%) from 4 consecutive cycles of the National Health and Nutrition Examination Survey (2007–2014) were included in this study after a rigorous screening process. Participants were stratified by their dietary magnesium intake into low-intake (<267 mg/day) and high-intake (≥267 mg/day) groups. We assessed differences between serum vitamin D levels and the risk of developing insulin resistance (interaction test), using a weighted multivariate logistic regression to analyze differences between participants with low and high magnesium intake levels.

**Results:**

There was a negative association between vitamin D and insulin resistance in the US adult population [OR: 0.93 (0.88–0.98)], *P* < 0.001. Dietary magnesium intake strengthened the association (*P* for interaction < 0.001). In the low dietary magnesium intake group, vitamin D was negatively associated with the insulin resistance [OR: 0.94 (0.90–0.98)]; in the high dietary magnesium intake group, vitamin D was negatively associated with insulin resistance [OR: 0.92 (0.88–0.96)].

**Conclusion:**

Among adults in the United States, we found an independent association between vitamin D level and insulin resistance, and this association was modified according to different levels of magnesium intake.

## Introduction

Insulin resistance (IR) refers to an efficiency decline in the performance of insulin to promote glucose uptake and utilization in tissues or cells (1, 2). It can be caused by a variety of reasons and pushes the body to compensate by producing too much insulin to keep the blood sugar within a normal range. IR is a core component of metabolic syndrome and type 2 diabetes (3–5). It may precede other cardiometabolic risk factors, as mentioned in the study of Gong et al. (6). Yin et al. also conducted a meta-analysis considering IR and the risk of thyroid cancer in 2018 and found that people with IR had a significantly greater chance of developing thyroid cancer (7). IR is also associated with the incidence of colorectal cancer (8, 9) and lung cancer (10, 11). In 2021, the International Diabetes Federation published guidelines stating that 629 million people aged 20–79 years are expected to develop type 2 diabetes by 2045 all over the world, for which one of the main causes is IR (7). In summary, to understand the risk factors underlying the development of health care is significant for the health of the World’s people.

Vitamin D (Vit D) is a lipid-soluble vitamin whose main function is to maintain the balance between calcium metabolism and bone formation in the human body. Vit D is activated in the body to acquire the biological activity of a hormone, with 1, 25-bishydroxyvitamin D3 acting as its main active form. According to related studies, Vit D level may be a protective factor against the development of IR (12–15). However, this conclusion is considered controversial at present. A cross-sectional analysis by Schleu et al. found that lower Vit D levels were strongly associated with increased IR in obese women (16). Szymczak-Pajor and Śliwińska (17) suggested that Vit D deficiency is one of the factors that accelerates the development of IR. In contrast, a randomized controlled trial by Gulseth et al. found that Vit D levels and IR were not correlated with one another (18) and similar findings were also reported by Margaret and Lansang (19). The reason for these variations may be that the adjustment strategies were not consistent across studies. Among them, we are particularly concerned about the absence of a certain factor in most of these studies: dietary magnesium intake.

Magnesium is the second most abundant divalent ion in cells following potassium ions, and it has been recognized as a cofactor in > 300 enzymatic reactions. It is essential for the modulation of blood pressure (20), insulin metabolism (21), and other physiological functions. Meanwhile, magnesium is closely related to Vit D synthesis, and previous studies have shown that magnesium is necessary for the movement and activation of Vit D in the blood (22). A randomized clinical trial published in 2018 by Dai et al. showed that magnesium optimizes Vit D status in the body with a bidirectional regulatory effect; in other words, magnesium can be optimized according to the body’s original Vit D level so that Vit D levels are maintained in the normal range (23). Further, all enzymes used for the metabolism of Vit D seem to require magnesium, which acts as a co-factor in the enzymatic reactions of the liver and kidneys (24). Magnesium intake alone or its interaction with Vit D intake may contribute to Vit D status (25, 26). The enzymatic activation of 25-hydroxylase in the liver and 1α-hydroxylase in the kidneys is a process that requires magnesium. Magnesium is also needed to deactivate Vit D when levels are too high (22). Previous studies presented that concentrations of cytochrome P450 (CYP) enzymes are modified by magnesium level (27). Cytochrome P450 enzymes include not only the Vit D–activating enzymes [i.e., 25-hydroxylase (e.g., CYP2R1) and 1α-hydroxylase (i.e., CYP27B1)] but also Vit D–deactivating enzymes [i.e., 24-hydroxylase (i.e., CYP24A1 and CYP3A4)]. 25-Hydroxylase synthesizes 25 (OH)D from Vit D3 or Vit D2 in the liver, and then 1α-hydroxylase synthesizes active 1, 25 (OH) 2D from 25 (OH)D in the kidney. 24-Hydroxylase metabolizes both 25 (OH)D and 1, 25 (OH) 2D to inactive forms: 24, 25-dihydroxyVit D and 1, 24, 25-trihydroxyVit D, respectively. Finally, CYP3A4 degrades 24, 25-dihydroxyVit D and 1, 24, 25-trihydroxyVit D (23). Other studies have shown that Vit D is transported through the body in combination with a carrier protein, i.e., Vit D-binding protein, and the activity of this protein is also dependent on magnesium (22). Thus, magnesium is a co-factor for Vit D biosynthesis, transport, and activation.

As we know magnesium is an activator of Vit D and also modulates IR, the question of whether magnesium can influence the link between Vit D and IR deserves consideration. To date, however, there has been little research on this issue. Therefore, we conducted a clinical study of the effect of magnesium intake on the relationship between Vit D and IR. In the present study, we hypothesized that magnesium ingestion could affect the association between Vit D and IR. The aim of this investigation was to explore the effect of magnesium intake on the relationship between Vit D and IR using a nationally representative public database in the United States in an effort to provide some reference for subsequent revelation of its mechanism of action.

## Materials and Methods

### Data Sources

This study was a large cross-sectional analysis using data from four cycles (2007--2014) of the National Health and Nutrition Examination Survey (NHANES).^1^ The NHANES project is a research project of U.S. citizens that uses a multi-stage stratified probability design with a collection sample representative of the overall sample of non-institutionalized U.S. citizens. These data include demographic data, food data, physical measurements, laboratory data, and questionnaire data. All NHANES-based studies are approved by the National Health Statistics Research Ethics Review Board. Ethical approval and more detailed information can be found on the NHANES Ethics Review Committee website^2^ (28).

### Study Design and Participant Population

This study was designed as a cross-sectional analysis. The target independent variable was the serum Vit D level recorded at the time that participants were tested. The dependent variable was whether the participant was diagnosed with IR. Grouping was done by median magnesium intake, with participants added to a low-intake (<267 mg/d) group (*n* = 2,436) or a high-intake (≥267 mg/d) group (*n* = 2,442).

A total of 40,617 participants completed interviews and examinations at the Mobile Examination Center (MEC) from 2007 to 2014. Participants with any of the following conditions were excluded from the current study: (1) age below 20 years (*n* = 17,944); (2) no serum Vit D testing (*n* = 2,017); and (3) missing insulin data or fasting glucose data (*n* = 15,778). Finally, a total of 4,878 participants were enrolled.

### Data Collection

All study data were collected by trained professionals and included demographics (age, sex, race, education, etc.), anthropometric measurements (height, waist circumference, weight, body mass index [BMI], etc.), health-related behaviors (smoking and exercise), and biochemical tests [fasting plasma glucose, oral glucose tolerance test (OGTT), etc.]. All information was collected and blood samples were drawn in an MEC; basic information was collated immediately and serum samples were managed in scientific storage, then sent to the Laboratory Sciences Division of the National Center for Environmental Health, the Centers for Disease Control and Prevention (CDC), and designated authorized institutions for analysis.

#### Measurement of Magnesium Intake

The magnesium intake protocol used in this study was the consensus reached during the regular NHANES workshops for expert assessment of the protocol (29). The 24-h food-recall method has previously been used to determine dietary intake in large cross-sectional studies. In this study, data on the first 24 h of magnesium dietary intake were collected through a dietary-recall interview at the MEC. Daily magnesium intake was classified as high or low intake based on the median value (267 mg/day).

#### Measurement of Vit D

Immediately after serum was collected at the MEC, it was stored frozen at -30°C and subsequently shipped uniformly to the CDC Environmental Health Laboratory in Atlanta, Georgia, for Vit D measurement. Vit D levels were defined as the sum of Vit D3 and Vit D2 concentrations. Laboratory analysis was performed by ultra-high performance liquid chromatography–tandem mass-spectrometry (30).

#### Identification of Insulin Resistance

In previous research, the Homeostatic Model Assessment for Insulin Resistance (HOMA-IR) index was recognized as a good indicator of IR (31). The HOMA-IR index is calculated as fasting glucose - insulin (μU/mL) × fasting glucose (mmol/L)/22.5. In a study involving IR in American adults, a HOMA-IR value of ≥ 2.73 was considered indicative of IR. Therefore, in the present study, IR was defined by a HOMA-IR value of ≥ 2.73 (32).

#### Definitions of Other Variables

To confirm diabetes, the measured fasting glucose level was multiplied by 0.056 (rounded to three decimal places) to convert the unit from mg/dL to mmol/L. Diabetes was diagnosed when the following conditions were met: fasting glucose level of ≥ 7.0 mmol/L, OGTT result of ≥ 11.1 mmol/L, physician diagnosis, self-reported diagnosis, or taking diabetes medication (33).

Participants who fit into any of the following race categories were included: Mexican–American, other Hispanic, non-Hispanic white, non-Hispanic black, or another race. Their education was divided into three categories: high school graduates, college graduates or higher. Smoking levels included current, former and never smokers. Those who had smoked ≥ 100 cigarettes or more in the past and reported smoking on a few days or every day at the time of the interview were considered current smokers, those who had smoked < 100 cigarettes in the past but were not currently smoking were considered ex-smokers, and those who had smoked < 100 cigarettes in the past were considered non-smokers. BMI was calculated using height and weight values. Weight was measured by the researchers using an electronic sports measurement device (Seca GmbH, Hamburg, Germany), which is accurate in millimeters. Body weight was measured by researchers using a digital scale (Toledo Scale; Mettler-Toledo, LLC, Columbus, OH, United States), and, after measurement, pounds were converted to kilograms. The formula used for BMI was: BMI = weight (kg)/height (m^2^). Finally, dietary data were obtained from a dietary retrospective interview set up to collect dietary information for the previous 24 h, including total dietary energy, Vit D, calcium, magnesium, protein, and fiber intakes.

### Statistical Methods

NHANES selects 5,000 people each year from a sampling frame of 15 different locations in all U.S. counties. Thus, its data have broad U.S. population band variability. To prevent bias and inaccurate estimation of results due to over-sampling of minority groups, we used one of the weight values officially recommended by NHANES, which means that all our subsequent studies were analyzed based on weighted models.

All data were analyzed using R version 4.1.2 (R Foundation for Statistical Computing, Vienna, Austria), with continuous variables represented by detailed sample descriptions with a mean confidence interval (CI) of 95%. Categorical variables were represented by counts and weighted percentages. Skewed distributions were based on median and Q1–Q3 values. Normal distributions were described by median and standard deviation values. Continuous variables were compared between groups using Student’s *t*-test or the Mann–Whitney *U*-test based on the normality of the distribution, and comparisons were made using Fisher’s exact probability method. Covariates were selected based on potential confounders that may be associated with Vit D and IR. Gender, age, race, smoking, BMI, obesity, dietary intake, physical activity, and education were selected as covariates based on a combination of previous literature, international standards, and relevant clinical experience. Multiple interpolation was used to fill in the missing covariates with the aim of maximizing statistical power and minimizing bias. In addition, sensitivity analyses were conducted to see if the resulting complete data differed significantly from the original data. These evaluations showed that the data after multiple interpolation did not differ significantly from the original data and were not statistically significant (*P* > 0.05). Therefore, all results of our multivariate analysis were based on the dataset developed after multiple interpolation according to Rubin’s criterion.

Three multivariate logistic regression models were developed to analyze the relationship between Vit D and IR in the sample at different magnesium intakes, and smooth fitted curves were constructed. *P* < 0.05 (two-sided) was considered to be statistically significant. We also developed three linear regression models to analyze the relationship between Vit D and the HOMA-IR index at different magnesium intake levels. A sensitivity analysis was completed to ensure the robustness of the analysis. We transformed Vit D into a categorical variable and calculated *P*-values for trends. The aim was to test the possibility of observing the presence of non-linearity between Vit D and IR when Vit D level was used as a categorical variable (34).

## Results

### Basic Information of the Study Population

**FIGURE 1:**
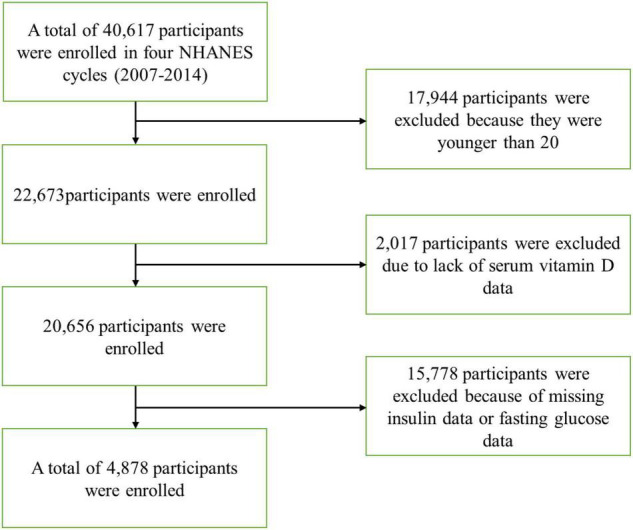
Flowchart of patient selection.

In the present study, a total of 4,878 participants from four NHANES cycles (2007–2014) were included (Figure 1). The basic information of the study population is detailed in Table 1. Grouping was performed based on magnesium intake, using a cutoff of 267 mg/d.

**TABLE 1:** Basic information description of participants.

		Dietary magnesium intake (mg/d)		
Variables	Total (*n* = 4,878)	<267 mg/d (*n* = 2,436)	≥267 mg/d (*n* = 2,442)	*P*-value
Age (year), mean ± SD	49.2 ± 17.7	50.3 ± 18.4	48.0 ± 16.9	<0.001
BMI (kg/m^2^), mean ± SD	28.9 ± 6.8	29.2 ± 7.0	28.6 ± 6.6	0.004
FPG (mmol/L), mean ± SD	6.0 ± 1.9	6.0 ± 1.9	5.9 ± 1.9	0.406
OGTT (mmol/L), mean ± SD	7.7 ± 4.3	7.9 ± 4.3	7.4 ± 4.2	<0.001
Serum-Vit D (nmol/L), median (IQR)	61.4 (44.2, 79.3)	58.6 (41.1, 77.0)	63.8 (48.0, 81.5)	<0.001
Sex, *n* (%)				<0.001
Male	2,353 (48.2)	947 (38.9)	1,406 (57.6)	
Female	2,525 (51.8)	1,489 (61.1)	1,036 (42.4)	
Race, *n* (%)				<0.001
Mexican–American	716 (14.7)	294 (12.1)	422 (17.3)	
Other Hispanic	477 (9.8)	243 (10)	234 (9.6)	
Non-Hispanic white	2,307 (47.3)	1,139 (46.8)	1,168 (47.8)	
Non-Hispanic black	913 (18.7)	544 (22.3)	369 (15.1)	
Other races	465 (9.5)	216 (8.9)	249 (10.2)	
Obesity, *n* (%)				0.001
No	3,134 (64.2)	1,510 (62)	1,624 (66.5)	
Yes	1,744 (35.8)	926 (38)	818 (33.5)	
Education, *n* (%)				<0.001
Did not graduate from high school	1,228 (25.2)	688 (28.2)	540 (22.1)	
Graduated from high school	1,068 (21.9)	600 (24.6)	468 (19.2)	
College education or above	2,582 (52.9)	1,148 (47.1)	1,434 (58.7)	
Activity, *n* (%)				0.772
Vigorous work activity	899 (18.4)	464 (19)	435 (17.8)	
Moderate work activity	1,032 (21.2)	505 (20.7)	527 (21.6)	
Walk or bicycle	682 (14.0)	343 (14.1)	339 (13.9)	
Vigorous recreational activities	330 (6.8)	168 (6.9)	162 (6.6)	
Moderate recreational activities	1,935 (39.7)	956 (39.2)	979 (40.1)	
Diabetes, *n* (%)				<0.001
No	3,905 (80.1)	1,892 (77.7)	2,013 (82.4)	
Yes	973 (19.9)	544 (22.3)	429 (17.6)	
Season of examination, *n* (%)				0.327
Winter	2,304 (47.2)	1,133 (46.5)	1,171 (48)	
Summer	2,574 (52.8)	1,303 (53.5)	1,271 (52)	
Dietary factors				
Energy (kcal)	2106.1 ± 10.3	1582.5 ± 6.7	2628.4 ± 15.1	<0.001
Protein (gm)	81.9 ± 42.9	59.1 ± 24.8	104.6 ± 45.0	<0.001
Fiber (gm)	16.7 ± 10.3	10.7 ± 5.1	22.7 ± 10.7	<0.001
Calcium (mg)	920.7 ± 603.4	639.1 ± 342.2	1201.6 ± 672.7	<0.001

*BMI, Body Mass Index; FPG, Fasting plasma glucose; OGGT, Oral Glucose Tolerance Test.*

The mean age of all participants was 49.2 ± 17.7 years. In the low-intake group, the mean age was 50.3 ± 18.4 years, while, in the high-intake group, the mean age was 48.0 ± 16.9 years, and the difference in age between the two groups was statistically significant (*P* < 0.001). The proportion of obese participants in the low-intake group increased (35.8 → 38%) and the proportion of the same in the high-intake group decreased (35.8 → 33.5%) over time, respectively. In the low-intake group, BMI, fasting plasma glucose, and OGTT levels were higher than those in the high-intake group. In contrast, Vit D levels were significantly higher in the high-intake group than the low-intake group.

### Bar Figure Analysis

**FIGURE 2:**
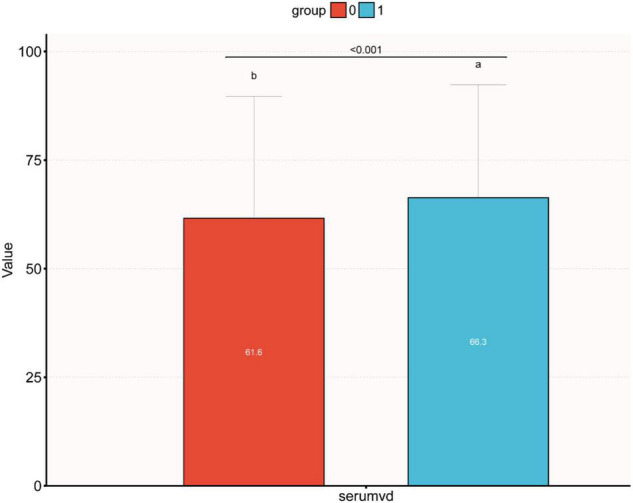
Bar figure of the differences between different vitamin D levels in the high and low dietary magnesium intake groups. Median vitamin D levels were significantly higher in the high magnesium intake group than in the low dietary magnesium intake group (0: low dietary magnesium intake group, 1: high dietary magnesium intake group, 66.3 vs. 61.6 nmol/L, *p* < 0.001).

**FIGURE 3:**
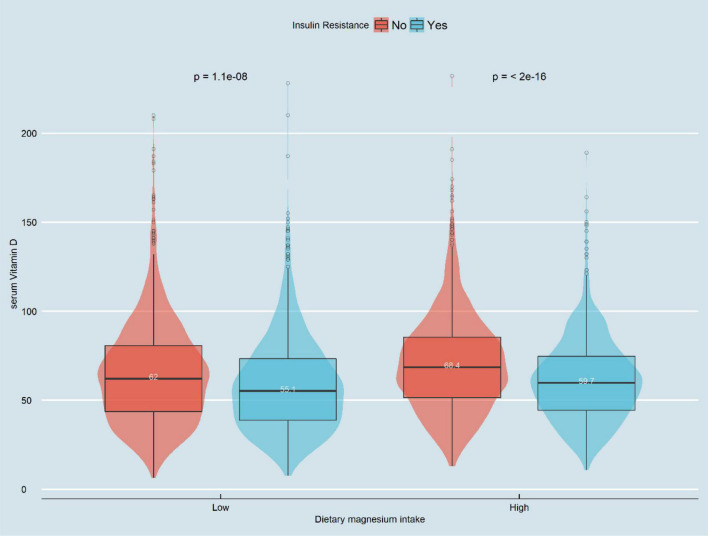
Violin chart of distribution of serum vitamin D in patients with IR group by magnesium intake. In the low-magnesium group, serum vitamin D levels in those with insulin resistance were significantly lower than those without insulin resistance (55.1 vs. 62.0 nmol/L, *P* < 0.001). In the high-magnesium group, serum vitamin D levels in those with insulin resistance were significantly lower than those without insulin resistance (59.7 vs. 68.4 nmol/L, *p* < 0.001).

Figure 2 displays the difference in Vit D level between high and low Mg intake groups (61.6 vs. 66.3 nmol/L, *P* < 0.001). Meanwhile, we observed that the Vit D level differed among the IR-positive and IR-negative groups (*P* < 0.001), as shown in Figure 3. The IR-positive group exhibited significantly lower Vit D levels than the IR-negative group (high Mg intake group: 59.7 *vs.* 68.4 nmol/L, *P* < 0.001; low Mg intake group: 55.1 vs. 62.0 nmol/L, *P* < 0.001).

### Univariate Analysis of Which Variables Might Be Associated With Insulin Resistance

Univariate logistics regression was used to detect which factors were associated with the occurrence of insulin resistance. As shown in Table 2, women had a lower probability of developing insulin resistance relative to men [OR: 0.8 (0.71–0.89)]. Compared to Mexican–Americans, other Hispanics [OR: 0.71 (0.56–0.9)], non-Hispanic whites [OR: 0.59 (0.5–0.7)] non-Hispanic blacks [OR: 0.72 (0.59–0.88)] and other races [0.43 (0.34–0.55)] had a lower probability of insulin resistance. age. factors such as obesity and IR were positively associated. In contrast, factors such as educational level, physical activity, Vit D and dietary magnesium intake were negatively associated with insulin resistance.

**TABLE 2:** Association of covariates and IR.

Variable	OR (95%CI)	*P*-value
Age	1.01 (1.01∼1.01)	<0.001
**sex, *n* (%)**		
Male	1	
Female	0.8 (0.71∼0.89)	<0.001
**Race/ethnicity, *n* (%)**		
Mexican–American	1	
Other Hispanic	0.71 (0.56∼0.9)	0.004
Non-Hispanic white	0.59 (0.5∼0.7)	<0.001
Non-Hispanic black	0.72 (0.59∼0.88)	0.001
Other races	0.43 (0.34∼0.55)	<0.001
**Obesity, *n* (%)**		
No	1	
Yes	6.73 (5.9∼7.67)	<0.001
**Education level, *n* (%)**		
Did not graduate from high school	1	
Graduated from high school	0.83 (0.7∼0.98)	0.026
College education or above	0.61 (0.54∼0.7)	<0.001
**Smoking status, *n* (%)**		
Current smoker	1	
Former smoker	0.95 (0.81∼1.13)	0.588
Never smoker	0.97 (0.84∼1.12)	0.669
**Physical activity, *n* (%)**		
Vigorous work activity	1	
Moderate work activity	0.97 (0.81∼1.16)	0.721
Walk or bicycle	1.13 (0.92∼1.38)	0.236
Vigorous recreational activities	0.93 (0.72∼1.2)	0.585
Moderate recreational activities	0.98 (0.83∼1.15)	0.774
**Season of examination, *n* (%)**		
Winter	1	
Summer	0.91 (0.81∼1.02)	0.099
Serum-Vit D	0.99 (0.99∼0.99)	<0.001
Mg-intake	0.99 (0.99∼0.99)	<0.001

*Data presented are ORs and 95% Cls.*

### Multivariable Logistics Regression Analysis of Vit D and Insulin Resistance

In the present study, three logistic regression models were constructed to analyze the independent association between Vit D level and IR and determine whether this association was influenced by different levels of magnesium intake. The model-based effect ratios (odds ratio [OR]) and 95% CIs shown in Table 3 indicate that each single-unit increase in Vit D was associated with a corresponding decrease in the probability of IR occurring. For example, in the unadjusted model, the total effect value was 0.99. Each single-unit increase in Vit D meant a 1% reduction in IR (OR 0.99; 95% CI 0.99–0.99). In the high-intake group, the effect ratio (OR) and 95% CI were 0.98 (0.99–0.99), respectively, while in the low-intake group, the effect-value ratio (OR) and 95% CI were 0.99 (0.99–0.99). In model 2, which was adjusted for sociodemographic data only, the overall effect-value ratio (OR) and 95% CI were 0.98 (0.97–0.99); in the high-intake group, the effect-value ratio (OR) and 95% CI were 0.96 (0.93–0.99); and in the low-intake group, the effect-value ratio (OR) and 95% CI were 0.98 (0.97–0.99), respectively. In the fully adjusted model 3, the overall effect-value ratio (OR) and 95% CI were 0.93 (0.88–0.98); in the high-intake group, the effect-value ratio (OR) and 95% CI were 0.92 (0.88–0.96); and in the low-intake group, the effect-value ratio (OR) and 95% CI were 0.93 (0.88–0.98), respectively. The above results suggest an independent association between Vit D level and IR and confirm that this association was influenced by different levels of magnesium intake.

**TABLE 3:** Interactive effect of vitamin D and dietary magnesium intake on IR (All participants).

	Model 1			Model 2			Model 3		
Variable	OR (95%CI)	*P*-value	*P* for interaction	OR (95%CI)	*P*-value	*P* for interaction	OR (95%CI)	*P*-value	*P* for interaction
Vit D	0.99 (0.99∼0.99)	<0.001		0.98 (0.97∼0.99)	<0.001		0.93 (0.88∼0.98)	<0.001	
**Magnesium intake group**									
<267 mg/day Vit D	0.99 (0.99∼0.99)	<0.001	<0.001	0.98 (0.97∼0.99)	<0.001	<0.001	0.94 (0.90∼0.98)	<0.001	<0.001
≥267 mg/day Vit D	0.98 (0.97∼0.99)	<0.001		0.96 (0.93∼0.99)	<0.001		0.92 (0.88∼0.96)	<0.001	

*Model 1: non-adjusted. Model 2: adjusted age, sex, race. Model 3: adjusted age, sex, race, obesity, education level, physical activity, smoking status, the season of examination, and dietary calcium intake.*

Three linear regression models were constructed to analyze the independent association between the Vit D level and HOMA-IR index and determine whether this association was influenced by different levels of magnesium intake. The model-based effect value β and 95% CI shown in Table 4 indicate that the Vit D level and HOMA-IR index were independently correlated and influenced by different levels of magnesium intake after adjusting for covariates according to the full model (Model 3). The overall effect value β and 95% CI were −0.04 (−0.06 to −0.02), respectively. Additionally, in the high-intake group, the effect β and 95% CI were −0.05 (−0.06 to −0.03), while in the low-intake group, the effect values β and 95% CI were −0.04 (−0.06 to −0.02).

**TABLE 4:** Interactive effect of vitamin D and dietary magnesium intake on HOMA-IR (All participants).

	Model 1			Model 2			Model 3		
Variable	β (95%CI)	*P*-value	*P* for interaction	β (95%CI)	*P*-value	*P* for interaction	β (95%CI)	*P*-value	*P* for interaction
Vit D	−0.02 (−0.02 to −0.01)	<0.001		−0.03 (−0.04 to −0.02)	<0.001		−0.04 (−0.06 to −0.02)	<0.001	
**Magnesium intake group**									
<267 mg/day Vit D	−0.02 (−0.02 to −0.01)	<0.001	<0.001	−0.03 (−0.04 to −0.02)	<0.001	<0.001	−0.04 (−0.06 to −0.02)	<0.001	<0.001
≥267 mg/day Vit D	−0.03 (−0.04 to −0.02)	<0.001		−0.04 (−0.05 to −0.03)	<0.001		−0.05 (−0.06 to −0.03)	<0.001	

*Model 1: non-adjusted. Model 2: adjusted age, sex, race. Model 3: adjusted age, sex, race, obesity, education level, physical activity, smoking status, the season of examination, and dietary calcium intake.*

### Linear Association Between Vit D and Insulin Resistance

**FIGURE 4:**
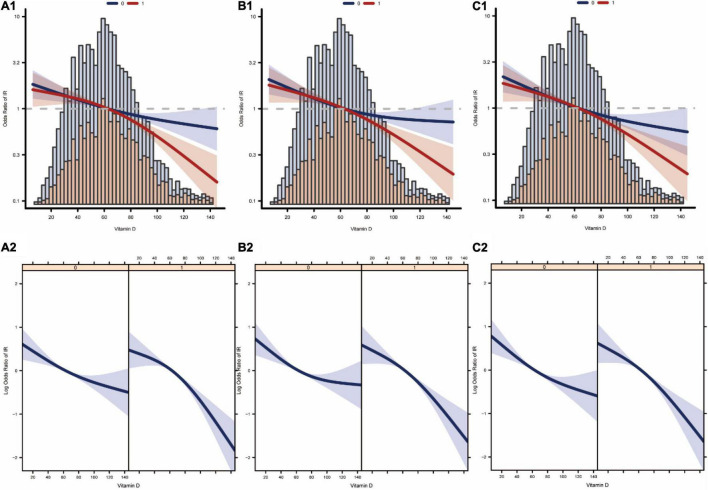
Curve fitting of vitamin D levels and the risk of developing insulin resistance. **(A1,A2)** Are association between vitamin D and insulin resistance at different levels of dietary magnesium intake after adjustment by model 1. **(B1,B2)** Are association between vitamin D and insulin resistance at different levels of dietary magnesium intake after adjustment by model 2. **(C1,C2)** Are association between vitamin D and insulin resistance at different levels of dietary magnesium intake after adjustment by model 3 (0: low dietary magnesium intake group, 1: high dietary magnesium intake group).

We analyzed whether there was a linear relationship between Vit D and IR at different levels of magnesium intake. Figures 4A1,A2 show the association between Vit D and IR after logistic regression without adjusting for latent variables. Figures 4B1,B2 show the association between Vit D and IR after logistic regression adjusted for model 2. Figures 4C1,C2 show the association between Vit D and IR after logistic regression adjusted for the full model (model 3). In summary, the associations between Vit D level and the risk of IR occurrence were linear at different levels of magnesium intake.

## Discussion

In recent decades, as the global economy has grown rapidly, the number of patients with type 2 diabetes has shown a significant increase in both developing and developed countries (35). Not only does this place a great burden on the world’s medical resources, its complications also greatly plague patients physically and psychologically. IR is one of the main causes of type 2 diabetes, and about 90% of cases are caused by IR. Aside from the rising incidence of diabetes, there are far more people suffering from IR. How to effectively prevent IR in advance before the progression to diabetes has also become a matter of great concern to researchers.

In this study, multiple linear regression models were developed to investigate whether there was a significant independent association between Vit D and the HOMA-IR index and to analyze whether the independent association between Vit D and the HOMA-IR index was affected by different dietary magnesium intake levels. In addition, multiple logistic regression models were developed to analyze the independent association between Vit D and IR and to assess whether the independent association between Vit D and IR was affected at different dietary magnesium intake levels. After excluding the potential confounding effects, the results showed that Vit D levels were independently correlated with the HOMA-IR index and IR in the multivariate regression analysis, and this association seemed to be influenced by different levels of magnesium intake. Smoothed fitted curves also showed a linear relationship between Vit D level and HOMA-IR index at different levels of magnesium intake. The probability of the occurrence of IR decreased alongside increasing Vit D levels. As shown in Figure 4, the association between serum vitamin D levels and insulin resistance was stronger in the high-magnesium diet group. As vitamin D levels increased, the incidence of insulin resistance decreased more significantly. This trend is evident in all models.

It has been reported that reduced plasma Vit D levels may produce excessive white adipose tissue, leading to IR and dyslipidemia (36). In a cross-sectional analysis conducted by Bilge et al. (37) in 2015 on a Turkish population of 39 individuals with normal weights and 66 individuals categorized as obese, Vit D was found to be negatively associated with the modified HOMA-IR index after adjusting for laboratory indicators, physical measurements, and other factors Research conducted by Wang et al. (38) also found that Vit D deficiency may lead to increased activity of the nuclear factor kappa-light-chain-enhancer of activated B-cells pathway, which promotes inflammation and leads to IR. These studies also support our results.

Magnesium is the second most abundant intracellular divalent cation after potassium ion, and it has been recognized as a cofactor in > 300 enzymatic reactions, with it being particularly essential for adenosine triphosphate metabolism (39). Low magnesium (2+) levels lead to defective tyrosine kinase activity, and insulin acts on receptors that are later damaged, altering cellular glucose transport and reducing cellular glucose utilization, thereby promoting peripheral IR in type 2 diabetes (21, 40). In addition, our finding is also consistent with another cross-sectional analysis conducted in the NHANES that magnesium intakes interact with serum Vit D levels in relation to type 2 diabetes (36). Thus, magnesium supplementation has the potential to increase Vit D activity such that it increases Vit D’s protection of pancreatic β-cells. A randomized clinical trial published in 2018 by Dai et al. showed that magnesium optimizes Vit D status in the body with a bidirectional regulatory effect; in other words, magnesium can be optimized according to the body’s original Vit D level so that Vit D levels are maintained in the normal range (23). Further, all enzymes used for the metabolism of Vit D seem to require magnesium, which acts as a co-factor in the enzymatic reactions of the liver and kidneys (24). Magnesium intake alone or its interaction with Vit D intake may contribute to Vit D status (25, 26). The enzymatic activation of 25-hydroxylase in the liver and 1α-hydroxylase in the kidneys is a process that requires magnesium.

A recent randomized trial found that magnesium treatment greatly reduced imidazole propionate, a microbial metabolite of histidine, compared to the placebo group. Imidazole propionate induces IR, and levels of imidazole propionate were higher in patients with prediabetes and type 2 diabetes. In addition to imipramine propionate, the same randomized trial found that magnesium treatment increased circulating levels of propionic acid and reduced levels of glutamate, two microbial metabolites. In fact, propionic acid and glutamate were associated with a reduced and increased risk of type 2 diabetes, respectively, and were inversely and positively associated with IR. In conclusion, the possible mechanism is that high magnesium intake increases Vit D synthesis on the one hand and improves microbial production of the three amino acid metabolites on the other hand, which in turn reduces IR and the risk of type 2 diabetes (41).

Although the current study is a cross-sectional analysis, our finding is consistent with that in a 2013 prospective cohort study in which the inverse associations between serum Vit D concentrations and risk of mortality due to cardiovascular disease only appeared in those with higher intakes of magnesium, but not in those with lower intakes of magnesium (25).

The present study has some limitations. First, because the NHANES database does not include some specific groups, such as pregnant women and children, it is uncertain whether the results of this study are applicable to these groups. We will analyze these groups in forthcoming studies, therefore this limitation will be addressed in the future. Second, the present study was a cross-sectional investigation, and no causal relationship could be drawn between Vit D levels and the HOMA-IR index as well as IR; thus, further cohort studies are needed to analyze this causal relationship. Finally, our dietary data were obtained from self-reported 24-h dietary-recall interviews and is therefore inevitably subject to some degrees of recall and self-report bias. However, the level of this influence is low and not sufficient to affect our results. This is because NHANES uses professional staff for data collection and a multistage stratified probability design approach for subject selection to decrease such bias. However, the present study also has certain advantages over other studies. In this study, a more comprehensive and more representative sample of participants was selected, with a unique representation of the entire U.S. population. In this study, the missing data were processed using statistical methods that are currently recognized by experts as more scientific (multiple interpolation) to maximize the statistical efficacy of the results as well as minimize the bias. Finally, smoothed fitted curves for Vit D and IR with different dietary magnesium intake levels were plotted to make the results more intuitive.

## Conclusion

In this study, Vit D levels were found to be independently associated with the HOMA-IR index and IR among American adults after adjusting for potential confounders, and magnesium intake strengthened this association. The results of this study provide new clinical insights. However, because this was a cross-sectional analysis that could not determine the role of magnesium in the association of Vit D levels with IR and HOMA-IR index, more randomized controlled studies or cohort studies are required to provide evidence in the future.

## Data Availability Statement

The original contributions presented in the study are included in the article/supplementary material, further inquiries can be directed to the corresponding author/s.

## Author Contributions

YL and RG conceived the idea and wrote the manuscript. HM, SC, JS, JQ, and YP collected, read the literature, and revised the article. JA and ZS read through and corrected the manuscript. All authors contributed to the article and approved the submitted version.

## Conflict of Interest

The authors declare that the research was conducted in the absence of any commercial or financial relationships that could be construed as a potential conflict of interest.

## Publisher’s Note

All claims expressed in this article are solely those of the authors and do not necessarily represent those of their affiliated organizations, or those of the publisher, the editors and the reviewers. Any product that may be evaluated in this article, or claim that may be made by its manufacturer, is not guaranteed or endorsed by the publisher.

## Footnotes

1
https://www.cdc.gov/nchs/nhanes/

2
https://www.cdc.gov/nchs/nhanes/irba98.htm
